# The gut microbiome of horses: current research on equine enteral microbiota and future perspectives

**DOI:** 10.1186/s42523-019-0013-3

**Published:** 2019-11-13

**Authors:** Anne Kauter, Lennard Epping, Torsten Semmler, Esther-Maria Antao, Dania Kannapin, Sabita D. Stoeckle, Heidrun Gehlen, Antina Lübke-Becker, Sebastian Günther, Lothar H. Wieler, Birgit Walther

**Affiliations:** 1 Advanced Light and Electron Microscopy (ZBS-4), Robert Koch Institute, Seestraße 10, 13353 Berlin, Germany; 2 Microbial Genomics (NG1), Robert Koch Institute, Berlin, Germany; 3 Research Data Management (MF4), Robert Koch Institute, Berlin, Germany; 4 Equine Clinic, Surgery and Radiology, Freie Universität Berlin, Berlin, Germany; 5 Institute of Microbiology and Epizootics, Centre for Infection Medicine, Freie Universität Berlin, Berlin, Germany; 6 Pharmaceutical Biology Institute of Pharmacy, Universität Greifswald, Greifswald, Germany; 7 Robert Koch Institute, Berlin, Germany

**Keywords:** Horse, Microbiome, Gastrointestinal tract, Microbiota, Disease, Health

## Abstract

Understanding the complex interactions of microbial communities including bacteria, archaea, parasites, viruses and fungi of the gastrointestinal tract (GIT) associated with states of either health or disease is still an expanding research field in both, human and veterinary medicine. GIT disorders and their consequences are among the most important diseases of domesticated Equidae, but current gaps of knowledge hinder adequate progress with respect to disease prevention and microbiome-based interventions. Current literature on enteral microbiomes mirrors a vast data and knowledge imbalance, with only few studies tackling archaea, viruses and eukaryotes compared with those addressing the bacterial components.

Until recently, culture-dependent methods were used for the identification and description of compositional changes of enteral microorganisms, limiting the outcome to cultivatable bacteria only. Today, next generation sequencing technologies provide access to the entirety of genes (microbiome) associated with the microorganisms of the equine GIT including the mass of uncultured microbiota, or “microbial dark matter”.

This review illustrates methods commonly used for enteral microbiome analysis in horses and summarizes key findings reached for bacteria, viruses and fungi so far. Moreover, reasonable possibilities to combine different explorative techniques are described. As a future perspective, knowledge expansion concerning beneficial compositions of microorganisms within the equine GIT creates novel possibilities for early disorder diagnostics as well as innovative therapeutic approaches. In addition, analysis of shotgun metagenomic data enables tracking of certain microorganisms beyond species barriers: transmission events of bacteria including pathogens and opportunists harboring antibiotic resistance factors between different horses but also between humans and horses will reach new levels of depth concerning strain-level distinctions.

## Equine microbiota and microbiomes: what we know so far

Humans and animals have a unique set of diverse microorganisms, an individual fingerprint. The complex and multi-levelled interactions between these resident microorganisms with respect to disease risks, health preservation, immunity and therapeutic possibilities are currently expanding research fields in both, human– and veterinary medicine. The intestinal tract of Equidae contains a diverse community of microorganisms that consists of fungi, parasites, protozoa, archaea, viruses and bacteria [1]. This entirety of different microorganisms associated with a distinct space is known as the microbiota, while the corresponding entity of genetic material is referred to as microbiome [2]. While this particular distinct and individual composition of a broad range of microorganisms includes essential nutrition suppliers and immune response supporters [3], it also contains taxa capable of causing disease [4]. All Equidae belong to a family of herbivorous mammals that possess a certain hindgut (caecum and colon) microbiota, enabling forage utilization for optimal nutrition. These microbes provide a substantial proportion of the horses’ daily energy needs through the fermentation of plant material to short chain fatty acids such as acetate, propionate, and butyrate [5, 6]. Consequently, gastrointestinal disturbance in the equine microbiota can result in alteration of fermentation patterns and, ultimately, metabolic disorders [7]. While knowledge about the role of archaea, viruses and eukaryotes residing within the GIT and their contribution to a healthy human microbiome is limited [8], even less data is available for horses, mirrored only by a few studies as shown in Table 1.

**Table 1:** Microorganisms with nourishment-associated activity in the gastro enteral tract of horses

Kingdom	Family	Genus	Species	Putative effects	Ref.
Bacteria	*Ruminococcaceae*	*Ruminococcus*	spp.	cellulolytic, fibrolytic bacteria	[167, 168]
			*favefaciens*	plant wall degradation	[169]
			*albus*	plant wall degradation	[169, 170]
	*Fibrobacteraceae*	*Fibrobacter*	*succinogenes*	monosaccharide and glycoside degradation	[169–172]
			*intestinalis*	plant wall degradation	[171]
	*Streptococcaceae*	*Streptococcus*	spp.	amyloytic^a^	[173]
			*bovis/equinus*	L-lactate producer	[174]
	*Lactobacillaceae*	*Lactobacillus*	*salivarius/ mucosae*	L-lactate producer, decarboxylating amino acids, vascoactive amines	[174], [137]
			*bulgaricus/ delbrueckii*	L-lactate producer	[174]
			*crispatus*	lactic acid bacteria	[175]
			*johnsonii*	lactic acid bacteria	[175]
			*reuteri*	lactic acid bacteria	[175]
			*equigenerosi*	lactic acid bacteria	[176]
			*hayakitensis*	lactic acid bacteria	[176]
			*buchneri*	lactic acid bacteria	[176]
			*vitulinus*	lactic acid bacteria	[176]
	*Acidaminococcaceae*	*Mitsuokella*	*jalaludinii*	D-lactate producer	[174]
		*Phascolarctobacterium*	spp.	fibre fermenters^b^	[168]
	*Veillonellaceae*	*Veillonella*	*gazogenes/ alcalescens*	lactat utilizing bacteria	[177]
	*Lachnospiraceae*	*Butyrivibrio*	spp.	cellulolytic, fibrolytic^c^	[167]
			*fibrosolvens*	amylolytic	[173]
		*Blautia*	spp.	fibre fermenters	[168]
	*Clostridiaceae*	*Clostridium*	spp.	cellulolytic, fibrolytic^d^	[167]
	*Eubacteriaceae*	*Eubacterium*	spp.	cellulolytic, fibrolytic	[167]
	*Prevotellaceae*	*Prevotella*	spp.	fibre fermenters	[168]
	*Succinivibrionaceae*	*Ruminobacter*	*amylophilus*	amylolytic	[173]
	*Enterococcaceae*	*Enterococcus*	*faecalis*	amylolytic	[173]
Fungi				fiber degradation	[178]
	*Neocallimastigaceae*	*Piromyces*	*equi*	cellulose degradation	[179]
Protozoa				hemicellulose, pectin degradation	[99]
Bacterio-phages				regulating bacterial species distribution	[180]
Archaea				methanogens^f^	[80], [81]

Within their enteral tract, horses are able to host up to 10^15^ bacterial cells [9] with the majority of bacteria residing in the colon, especially within the comparatively enlarged caecum [10]. The degradation of non-digestible cellulosic and hemi-cellulosic forage components by these microorganisms is crucial for the bioavailability of energy and other essential nutritional needs in horses [9].

Several diseases including cardiovascular disorders [11, 12], inflammatory bowel disease [13], diabetes [14–16], rheumatoid arthritis [17], depression [18] and progression of cancer [19–22] have, among others, been associated with distinct changes in human intestinal microbiomes in recent years. Compositional changes of the equine microbiota were similarly investigated with respect to its impact on certain diseases such as equine grass sickness [23], colitis and laminitis [24–26]. Moreover, the effects of distinct diets and dosage forms have been studied in elderly horses and horses in training [27, 28]. In the years that followed, maps of the equine microbiome [29–31] and the putative impact of probiotics such as *Lactobacilli* and *Bifidobacteria* were explored [32, 33]. Another recent focus of research is to unveil the putative composition of an equine hindgut “core” microbiota. This core microbiota should mirror the stable, consistent bacterial components including key microorganisms and their functions [30, 34–36]. In yet another study, the impact of antimicrobial treatment and anesthesia was investigated with respect to their role in shaping equine microbial composition [37, 38].

In this review we aim to provide an overview about the **i**) techniques used or available for equine microbiome exploration **ii**) current knowledge on equine hindgut microbiota with an emphasis on bacterial components **iii**) traits and factors which might influence equine microbiome diversity and composition and **iv**) future trends and perspectives in this field.

## How to study microbial communities: techniques currently available to define the equine enteral microbiome

**Fig. 1:**
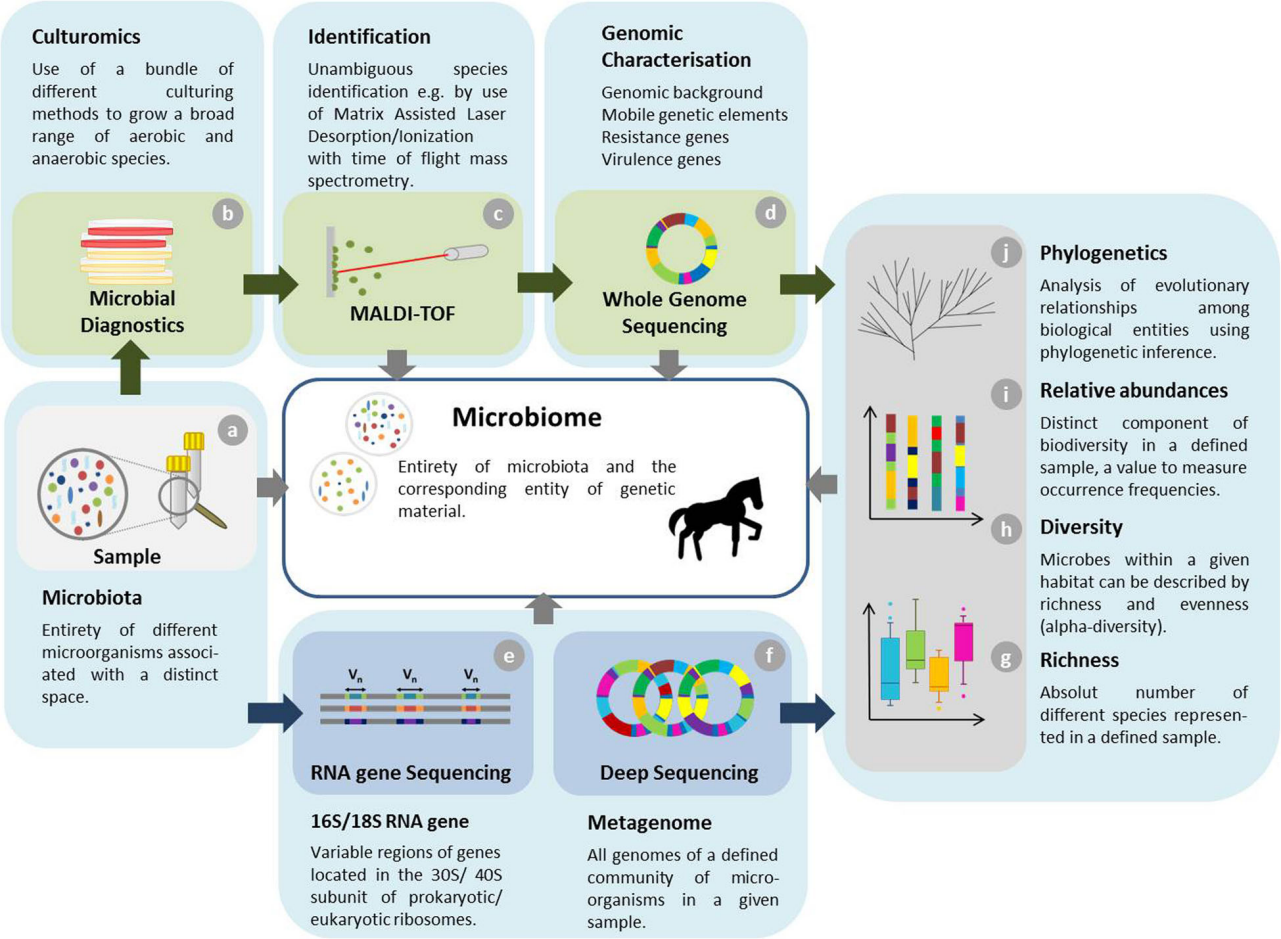
Workflow and synergistic application of differt techniques to study enteral microbiomes. Integrative and synergistic workflow to study equine microbiomes starting with dividing the fresh sample (**a**) for two general processing’s, microbial diagnostic in terms of culturomics (**b**-**d**) [157] and DNA sequencing approaches (**e** and **f**) for population analysis (**g**-**i**). A broad range of different aerob and aerobic culture conditions are used to initiate growth for microbial diagnostic (**b**), followed by rapid species identification by MALDI-tof mass spectrometry (**c**). Genome sequencing (**d**) allows (novel) species identification in case MALDI-tof provided no confident result or if resistance- and virulence encoding genes [158, 159] or other factors are of particular interest within a species. Both information sources allow identification of bacterial species present in the horse microbiota and their growth conditions. The second part of the sample should be stored native at − 80 °C until DNA extraction starts for either sequencing of variable regions of 16S/18S rRNA gene (**e**) allowing characterizing and quantifying taxonomic entities or sequencing of all genomes (metagenome) present in a sample (**f**). Further bioinformatics include description of richness (**g**), diversity indices (**h**) [160–162], relative abundances (**i**) and phylogenetics (**j**). Combination of classical diagnostics on a large scale and different techniques available to generate genomic data enable deep insights into microbiome composition and characteristics [163]

For interpretation of studies on the microbiome composition, including those of hindgut fermenters such as horses (Additional file 1), it is necessary to understand the different technologies currently used for data generation and exploration. Until recently, the identification of intestinal microorganisms was performed by culture-dependent methods limiting the outputs to cultivable species only [39]. These methods are, however, slowly being replaced and/or complemented by new comprehensive approaches such as “Culturomics”, a method which includes multiple growth conditions to a subdivided original sample together with extended incubation times. In combination with rapid identification methods for bacteria such as Matrix-Assisted Laser Desorption Ionization–Time of Flight Mass Spectrometry (MALDI-TOF-MS), a fast and extended overview on cultivable bacterial components of a sample of interest is possible. Mass spectra of so far unidentified species could be generated and assigned by the additional use of 16S rRNA sequencing [40]. Consequently, Culturomics can be seen as a kind of “rebirth” of culture-based techniques in microbiology [41], producing results which are easy to combine with other methods commonly used to study animal microbiomes (Fig. 1).

Overall, high-throughput sequencing approaches are currently the most predominant techniques to investigate microbiomes, in clinical research as well as in environmental science [42, 43]. The recent developments in DNA sequencing technologies, also referred to as next-generation sequencing (NGS), now allow researchers to study complex biological samples based on sequence information on a large scale [44]. In general, DNA is first purified from the samples and DNA sequencing is then used to characterize the associated taxa, employing either a ubiquitous marker gene such as the 16S rRNA gene for bacteria, the 18S rRNA gene for eukaryotes or an internal transcribed spacer (ITS) DNA present between rRNA genes for fungi. Alternatively, all DNA in a given sample is sequenced by use of shotgun metagenomics sequencing [45]. Since NGS allows for cost-effectiveness, sufficient resolution and sequencing depth for many research questions, this is one of the most commonly used techniques in medical- (food)hygiene- and environmental metagenomics studies [39].

One method to explore microbial compositions is NGS of the bacterial ubiquitous ∼ 1500 base pair 16S rRNA gene made up of nine hypervariable regions flanked by conserved sequences [46]. Here, primers are used to define resulting amplicons covering the hypervariable regions which then differ in amount and base composition per sample under investigation. Based on the nucleotide sequence similarity, these sequences are clustered into Operational Taxonomic Units (OTU) [47].

To ascribe taxonomic identities of a certain bacterial community, NGS results are compared to 16S rRNA gene sequence databases available, including Greengenes [48] and Silva [49]. With its conserved and variable sequence regions evolving at very different rates, the 16S rRNA sequences provide reliable data for investigating both close and distant phylogenetic relationships, and allow a precise assessment of phylogenetic relatedness of organisms [50]. Currently, a broad panel of bioinformatic tools designed for sequencing data analysis are available, including many which are open source and easy to operate [47]. Commonly used software to analyze 16S rRNA data from food/environmental samples include QIIME (Quantitative Insights into Microbial Ecology) [51], mothur [52], and USEAR (ultra-fast sequence analysis) [53]. These tools assign the sequences to specific taxonomic levels based on clustering for OTUs at different sequence identity thresholds.

However, there still are clear limitations when using NGS 16S rRNA based identification of bacteria beyond the family level [54], since current sequencing read lengths with Illumina technology only cover a region of around 460 bp mostly from the V3 and V4 region while a full-length or near full-length 16S rRNA sequence is needed for a confident taxonomic assignment of genus and species [50]. Since it is known that bacterial species differ with respect to their copy numbers of the 16S rRNA gene from one to 15 and more [55], amplification could lead to a bias considering semi quantitative proportions (relative abundances) in complex communities [56]. Moreover, the selection of primer sets used for amplification of the 16S rRNA gene might result in over- or underrepresentation of distinct bacterial species [57].

Shotgun sequencing of whole genome DNA samples provide the most complete information on the entire gene pool within a sample while the high amount of generated data requires substantial efforts of bioinformatics in sequence assembly, mapping and analyses [39]. In principle, the method is quite similar to those used for sequencing a single bacterial genome [58], but the output data consists of all genome sequences present in a given complex sample including archaea, bacteria, fungi and viruses. A recent study demonstrated that shotgun whole genome sequencing has multiple advantages compared with the 16S amplicon method such as enhanced detection of bacterial species, increased detection of diversity and abundance as well as increased prediction of genes relevant for example for antimicrobial resistance or virulence determination. In addition, providing sequence data of the whole genome of the present microorganisms in combination with whole genome reference databases greatly improved the accuracy of species detection [59]. A comprehensive overview on current methods frequently used for microbiome surveys together with means for beneficial complementation of different techniques and analysis methods is provided in Fig. 1.

However, creating valid results from shotgun sequencing of complex microbiomes is still challenging and computationally intensive [60]. Till date, open databases available to assign genomic data by mapping metagenomics reads provide more primary whole genome sequencing (WGS) data for reference- and pathogenic strains, while colonizing or non-pathogenic bacteria had less often been sequenced in the past [61]. Consequently, a significant proportion of shotgun sequences is dedicated to “microbial dark matter” of gut microbiomes, since suitable reference genomes of non-cultivable and/or non-pathogenic bacteria are not available for assignments [44]. In addition, methodical standardization and the development of specific pipelines for data analysis and –reproducibility are still an ongoing matter of discussion [62]. Microbiome research reliability and -development depend on reliable data at free disposal. In fact, providing raw sequencing data lacking corresponding sets of metadata hinders any attempt to reproduce the original study results [63]. As a consequence, databases like NCBI SRA (https://www.ncbi.nlm.nih.gov/sra) were established for storing and sharing sequencing data. Taken together, NGS technology developments have shown great progress in recent years, but technical issues still exist, mainly related to the need of continuously updated databases, specific bioinformatic tools, and functional correlations [62].

In 2012, first studies addressing the equine microbiome were published, reporting on 2–6 horses providing up to 16 specimens subjected to microbiome analysis. Since then, the numbers of animals under investigation, samples and data processing, as well as evaluation opportunities have increased dramatically. Additional file 1 provides a comprehensive overview on microbiome surveys in horses published so far (2018).

## Microbiomes’ markers: species abundances, sample richness and diversities

One of the most important goals of many microbiome surveys is to explore and describe differences in the relative abundances of bacterial taxa induced by environmental changes [64]. As the abundances generated by NGS technology are semi-quantitative by definition, the observed dynamics may not accurately reflect those of the actual taxon densities, a fact that was shown by way of comparison of single-cell counting by use of flow cytometry with 16S rRNA sequences [64].

To measure and analyze variation and composition of microbial communities, indices describing diversity have been implemented. In 1960, alpha- and beta diversity were defined, where the alpha diversity allows to estimate species number (richness) and distribution (evenness) within a particular sample, while a beta diversity measure acts like a similarity score between populations of different samples [65]. Since then, several different diversity indices have been defined [66]. Among the most commonly used diversity indices are taxon based approaches, Simpson’s index [67], Coverage (C) [68], Chao1 richness estimator [69], Shannon index [70] and Shared OTUs [71–73]. To date, at least 15 different tools for taxonomic profiling are available for metagenomics, already compared and benchmarked by use of various datasets [60].

## Current understanding of the equine microbiome

For all mammalian species, scientific evidence points towards a strong relationship between enteral microbiome composition and its function [74]. Considering data available on composition of microbial communities residing in different animal species’ guts, current knowledge exposes a clustered gastrointestinal microbiome according to differences in their gut microbiota for all carnivores, herbivores and omnivores [75]. For instance, nourishment based on animal proteins results in an increased number of *Firmicutes* among the respective microbiota while, in contrast, plant based diets result in more fibers and those microbiomes yield an increased number of *Bacteroidetes*, cellulose- and xylan degrading bacteria [28]. Recent studies revealed distinct individual ecosystems for each compartment of the equine gut, with more similarities regarding composition of microbiota in neighboring compartments than between more distant ones [30]. At present, two main regions need to be distinguished: the upper- and the lower GIT [29]. By way of comparison, the upper equine gut (stomach, jejunum and ileum) shows a more variable microbiota substantiated due to a high throughput of environmental bacteria present in the forage. Moreover, members of the α*-Proteobacteria* such as *Methylobacterium* sp., *Rhizobium* sp*.* and *Sphingomonas* sp. are commonly abundant in this gut region [29]. In contrast, composition of the microbiota residing in the lower GIT of horses (caecum and colon) seems remarkably stable, despite variables such as individual history, breed or age.

Beside a rich population including a diverse spectrum of bacterial species with their bacteriophages, the equine hindgut microbiota also encompasses protozoa, fungi, yeasts, and archaea [76]. Considering resident bacteria, *Firmicutes*, *Bacteroidetes* and *Verrucomicrobia* are amongst the predominating phyla in the equine hindgut [28, 30, 77–79]. Further studies revealed an abundant population of methanogenic archaea in the equine colon [76]. These microbes metabolize H_2_ and CO_2_ to produce methane [80] and probably support the degradation of cellulolytic bacteria in the lower gut [81, 82]. Metabolic pathways essential for sufficient nourishment of horses depend on functional interactions of mandatory microbes needed for a successful degradation of nutrients. Some bacterial families belonging to the resident phyla as well as other microorganisms of the equine GIT have been characterized with respect to their (predicted) nourishment-associated activity (Table 1).

Activity of microorganisms leading to changes within gastrointestinal microbiota in horses. Further proposed effects of distinct microorganisms are indicated by small letters. Abbreviations: Ref., Reference; ^a^, generates neurotransmitter serotonin (5-hydroxytryptamine, 5-HT) [83]; ^b^, associated with succinate pathway for production of short chain fatty acid propionate [84]; ^c^, butyrate producers [85], butyrate shows protective function for colonocytes [86], ^d^, major producers of short chain fatty acids [87]; ^e^, possesses coding region for major exoglucanase [88]; ^f^, use of H_2_ and CO_2_ to produce methane, might boost the carbohydrate-degrading activity of cellulolytic bacteria [80, 81].

An important role in the enteral degradation of vegetal fibres was assumed for anaerobic fungi. In 2003, *Piromyces equi*, an anaerobic monocentric fungus, was reported to possess a major exoglucanase, which is fully capable of digesting cellulose [88, 89]. Next to *Piromyces equi* only two other morphological and metabolically different fungal species were described: *Piromyces citronii* and *Caecomyces equi* [9]. Evidence also exists for other novel fungal taxa grown from equine feces, which still need to be characterized and investigated further [90].

At present, knowledge is scarce concerning the role of bacteriophages in the equine gut. Several studies estimate a proportion of 10^10^ to 10^11^ bacteriophages per gram feces [91, 92], including up to 60 morphologically distinct phage types [93]. Golomidova et al. (2007) provided evidence of phage affinity for bacteria with high population numbers [92]. A dense population is commonly more embedded and adjusted in its biological environment than bacteria with a lower population number. The authors pointed out a direct link between diversity and abundance of *Escherichia coli* strains and the relative abundance of specific coliphages. Many ecological systems are shaped from predator-prey interactions. However, the GIT often promotes commensal relationships between different members of the community [94]. It is assumed that bacteriophages influence the fitness of intestinal bacteria and support colonization and host adaption, particularly in cases of environmental changes, including antibiotic forces [94–96]. Amongst others, Cann et al. have identified *Siphoviridae*, *Myoviridae*, *Podoviridae* and vertebrate *Orthopoxvirus* in horse feces, but 26% of viruses identified in that study were unclassified in 2005 [91].

Yet, the role of intestinal protozoa such as *Ciliates* [97, 98] is not well understood. A beneficial while only limited function in cellulose digestion and degradation of pectin seems likely [99, 100].

Age is among the most influencing factors of individual enteral microbiomes, while the initial microbiome already depends on the location of birth. In humans, even the type of birth (natural delivery or *sectio caesarea*) brings about differences with respect to initial microbiome composition [101].

**Fig. 2:**
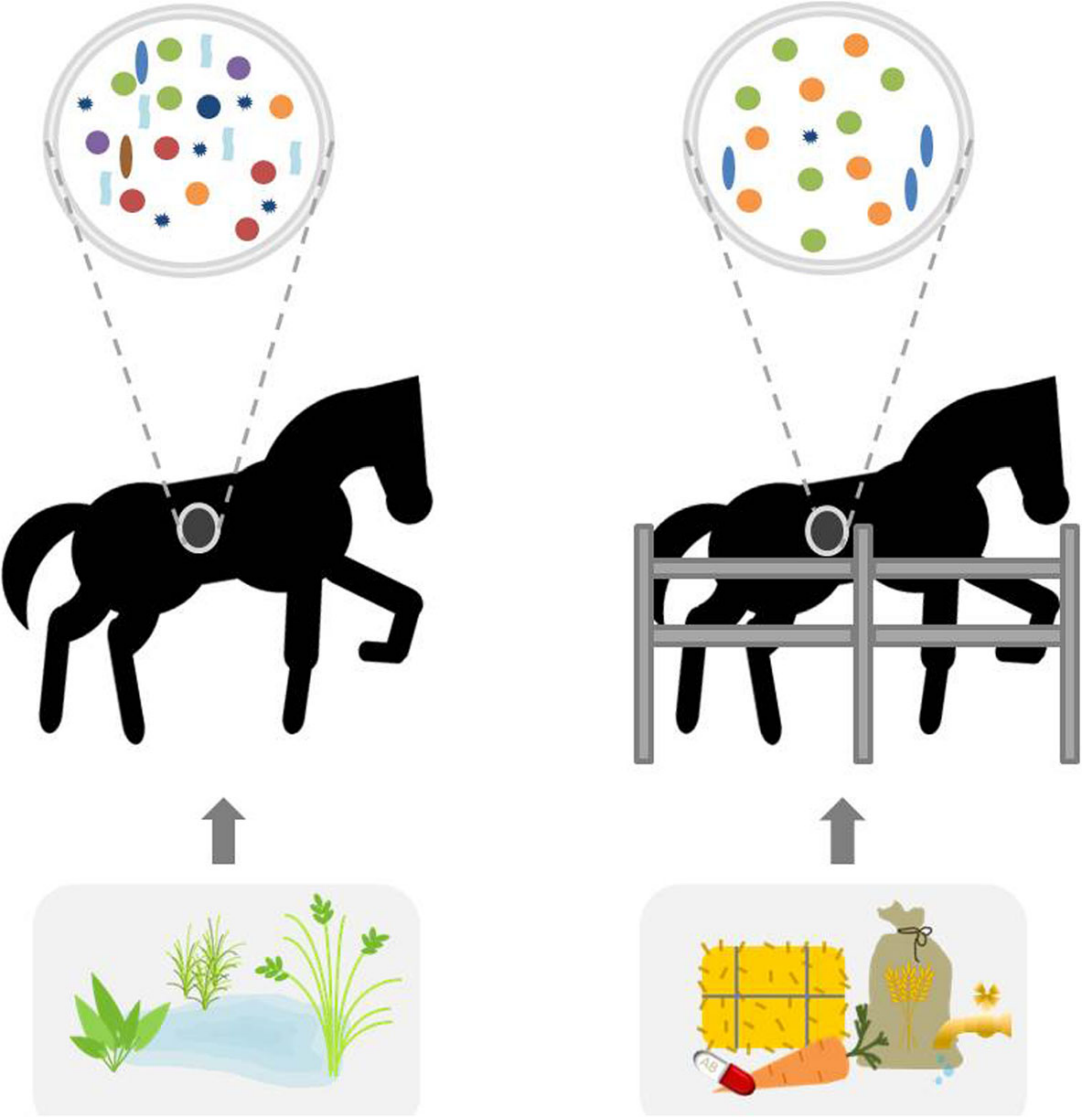
The anthropogenic impact on horse microbiomes. Microbiomes of non-domesticated horses (left) include a more diverse spectrum of microbiota compared to those of domesticated horses (right). Beyond age, differences in housing and pasture habits [164], composition of feeding diets [165, 166], contact with humans, veterinary health care and medication seem to be among the most influencing factors for equine microbiomes [101]. Interestingly, similar observations are available for the humans, since indigenous populations seem to have a much broader spectrum of microbiota compared with industrialized countries [104]s

While new born foals commonly have a rich and diverse microbiota with *Firmicutes* as predominant phyla [102, 103], foals between two and 30 days in comparison host a decreased level of different microorganisms, with *Verrucomicrobia* (e.g. *Akkermansia* spp*.*) predominating [102]. After 60 days, the microbiome consists of a relatively stable population, and microbiomes of 9 month-old foals only show few differences compared with those of adult individuals [102]. Considering levels of species diversity, microbiomes of older horses (19–28 years) once again show a decreased level with respect to the diversity of residing organisms [28]. A comprehensive overview about factors affecting GIT microbiome composition while affecting relative abundance of distinct microorganisms in horses is given in Table 2. Interestingly, the degree of domestication of *Equidae* under consideration seems to have an important impact on their enteral microbiome, which is summarized in Fig. 2. Free living individuals show a more diverse microbiome composition as their conspecifics in captivity [101], an observation which might mirror loss of diversity among human enteral microbiomes in more industrialized countries [104]. Horse domestication interferes with social structures like inter-individual relationships, shared environments and nourishment [101]. Comparative composition analysis of microbiomes of non-domesticated and domesticated horses living in the same area with similar plant diets revealed that fecal microbiomes of the latter group had a significantly lower abundance of the Clostridia genus *Phascolarctobacterium* for producing the short chain fatty acid propionate [101]. Moreover, microbiomes of non-domesticated horses harbor a significantly higher relative abundance of producers of enteric methane like *Methanocorpusculum archaea* [101], which may boost the carbohydrate-degrading activity of cellulolytic bacteria (Table 2).

**Table 2:** Effects of specific factors on equine intestinal organism abundances

Factor	Effect on organism abundance	Organisms in enteral microbiome	Reference
highly concentrated (grain) feed	increase	lactic acid bacteria, especially *Streptococcus spp*. and *Lactobacillus spp*.	[181] [31]
high-starch fed	increase	*Succinivibrio*	[28]
high-starch fed	decrease	*Clostridiales*, *Lachnospiraceae*	[28]
haylage	putative increase	*Fibrobacter succinogenes, Fibrobacter intestinalis*	[106]
grass-based diet	increase	*Bacteroidetes*, *Lachnospiraceae Bacillus,* *Lactobacillus, Streptococcus*	[181]
grass-based diet	decrease	*Fibrobacter*, *Ruminococcus*	[181]
high oil and high starch diets	increase	*Proteobacteria*	[28]
increasing age	increase	*Euryarchaeota, Actinobacteria, Bacteroidetes, Chlamydiae, Chloroflexi, Planctomycetes, Spirochaetes,* TM7*, Verrucomicrobia*	[182]
increasing age	decrease	*Proteobacteria, Gammaproteobacteria, Enterobacteriaceae, Enterococcus*	[182]
domestication	lower	*Methanocorpusculum*	[101]
pH below 6.0	decrease	*Ruminococcus albus*, *Fibrobacter succinogenes*	[6]
pH below 6.0	increase	*Streptococcus bovis, Lactobacillus spp., Mitzuokella spp.*	[6]
parasite egg burden	decrease	*Bacteroides, Clostridium XIVa, Ruminococcus, unclassified Lachnospiraceae*	[178, 183]
parasite egg burden	increase	*Clostridium* IV*, Coprococcus, Anaerovibrio, Agreia, Oscillibacter, Turicibacer,* unclassified *Cystobacteraceae, Campylobacter, Bacillus, Pseudomonas*	[178, 183]
laminitis	increase	*Lactobacilli*, *Escherichia coli*	[138, 184]

## Attempts to define the “core bacteria” of the equine microbiome

Microbial communities which commonly appear in all assemblages associated with a specific habitat are likely critical to the function of that environment [36]. Consequently, identifying of a defined core composition of microorganisms is an important step in defining a ‘healthy’ microbial community [36]. The core community at the Operational taxonomic Unit (OTU) level in feces is defined as “being present in all samples included in the study at 0.1% relative abundance (or greater)” [105]. Defining an essential core might be useful to predict the impact of perturbations and to preserve or restore a microbiome associated with a healthy condition [36]. Despite the unarguable individual composition of each horses’ microbiome [106] a so called “core microbiome” was declared including “key microbes” present in most individuals [7, 28, 30, 35]. Considering the vast diversity of intestinal bacteria known for ruminants, the equine gut microbiome seems to comprise a lower number of species as “core” population, with the richest diversity (33 bacterial families) residing in the right dorsal colon [7].

*Firmicutes* represent the largest phylum of the equine intestinal bacterial community ranging from 40% up to 90% in different compartments, including *Clostridia* and *Bacilli* [7, 30]. *Clostridiales* such as the aerobic *Lachnospiraceae* seem to be a part of the intestinal “core microbiome” in all mammals [28]. They produce butyrate which is known for its protective function of colonocytes [107]. Although the families *Ruminococcaceae* and *Fibrobacteraceae* represent only a small percentage of the bacterial community, both were considered as being part of the “core” along the entire equine hindgut [9]. These bacteria are involved in plant-wall degradation (Table 1) and their absence may influence the overall balance of the microbiome, therefore these cellulolytic bacteria were seen as “keystone species” [108].

The second largest group to address here are *Proteobacteria*, comprising a broad range of gram-negative bacteria, including Enterobacteriales and Pseudomonadales. The intestinal diversity of *Proteobacteria* is driven by the uptake from the environment, where these bacteria reside to certain abundances. Consistently, *Proteobacteria* are predominant in the upper part of the equine GIT [29], with highest abundance in the equine Ileum (including *Pasteurellales*) with approximately 33% [30]. In view of the overall diversity of residing *Proteobacteria,* various functional activities can be assumed, which are not entirely known yet. For instance, some members of *Proteobacteria* are known for their role in intestinal nitrogen fixation [109]. Nevertheless, an overabundance is reported to be associated with inflammatory intestinal diseases and dysbiosis like colic in horses [25, 110].

The third group consists of *Verrucomicrobia*. *Verrucomicrobia* is an abundant phylum within the environment, especially in soil [111]. *Verrucomicrobia* are part of the PVC superphylum, named for its member phyla *Planctomycetes*, *Verrucomicrobia* and *Chlamydiae, which* are distinct phyla of the domain bacteria proposed initially on the basis of 16S rRNA gene sequence analysis [112]. These bacteria are considerable residents in equine caecum, small colon, rectum and feces with relative abundance ranging from 10 to 23% [30]. *Verrucomicrobia* gained increasing attention in obesity and metabolic disease research in humans [113, 114]. Akkermansia, a mucin-degrading genus within the phylum Verrucomicrobia helps to maintain the integrity of the mucin layer and decreases bowel inflammation [115]. In summary, the overall diversity of the core bacterial community of domesticated horses seems to be surprisingly low, a fact that was discussed as a possible reason for the sensitivity of horses to GIT diseases [28].

## Diseases, drugs and feeding are associated with changes in the equine microbiome

Horses have a sensitive intestinal tract, and exercise [10], transport and fasting [38] ensure verifiable changes in the equine microbiome composition. A comprehensive overview on studies addressing composition and changes of the equine microbiome in healthy and diseased animals together with the techniques used by the individual study group is provided in Additional file 1. Important findings from these studies addressing major issues of microbiome research in horses will be explained and summarized in the following section.

Since an appropriate and balanced diet is essential for optimal successful degradation of nutrients and health in Equidae, incorrect feeding might induce dysbiosis or increases general vulnerability [31, 116]. Dysbiosis in microbiome composition was found to be associated with horses suffering from enteral disorders [25, 110].

A balanced system of intestinal microorganisms is an important health value, not surprising an unbalanced enteric microbiota could cause colitis [25]. Colitis refers to an inflammation of the gut mucosa of the large bowel (cecum and colon) which is either characterized by an acute or long-term process. Commonly, acute colitis is characterized by a sudden onset of profuse watery diarrhea. The fast and excessive loss of enteric fluids is able to induce death by dehydration or even hypovolemic shock [117]. Equine colitis can be triggered by multiple conditions including bacterial infections, infestation by parasites or antimicrobial treatment [117–119]. Bacteria-associated inflammation is commonly associated with *Salmonella* species, *Clostridioides difficile*, *Clostridium perfringens* and *Neorickettsia risticii* (Potomac horse fever) [120]. *Fusobacteria,* commonly rare in healthy horses, seem to be significantly enriched in case of diarrhea and colitis [25, 121]. Additionally, foals with diarrhea have shown a less rich microbiome composition in comparison with healthy foals together with decreased abundances for *Lachnospiraceae* and *Ruminococcaceae* [122].

It is difficult to pinpoint a precise cause for gut inflammation since further variables such as age, living space and individual case history of the horse influence the entire community of residing microbiota [117]. A common non-infectious cause of colitis in horses is receiving antimicrobials. Many reports have shown the association between antimicrobial treatment of horses and colitis [123, 124]. An imbalance of the fragile equine intestinal microbiota which may lead to bacterial overgrow seems to be inducible by a lot of antibiotics, including Penicillin [125], Cephalosporins [126] or Fluoroquinolones [127]. These antimicrobials have been associated with equine colitis [128], reflected by a significant transformation of the equine microbiome structure after consumption [37]. Costa et al. (2015) reported changes of equine fecal microbiota induced by trimethoprim-sulfadiazine, emphasizing a significant decrease of bacterial richness and diversity together with a drastic decrease of endosymbionts such as *Verrucomicrobia* [37]. Changes in the equine microbiome composition induced by antibiotics seemed to be specific for each drug and might therefore be predictable [37]. It seems to take 25 days to re-build the microbial composition back to individual baseline levels, but differences are still detectable beyond that time [37].

Moreover antimicrobial therapy is among the main risk factors for *Clostridioides difficile* associated colitis and colonization not only in humans but also in horses and other companion animals like dogs and cats [125, 129, 130]. Disruption of host microbiota homeostasis with reduction of microbiota density is most likely associated with reduced colonization resistance and may also contribute to a pro-inflammatory host immune response [131].

Colic is one of the most lethal diagnoses for horses which only 63% will survive [132]. Besides sand ingestion and colon displacement [117], further (stress) factors can be responsible for colic. Changes in feeding routine are also under suspicion for inducing rapid shifts in microbiome composition [133] and increased risk for colic [10, 134]. To identify microbiome changes strongly associated with colic [135], physiological changes in microbiomes of healthy horses need to be explored [106]. At present, there is a lack of data addressing the role of particular microbiome changes for the development of the equine colic syndrome.

Receiving anesthesia seems to be a putative further factor able to cause changes of the equine microbiome structure. Shifts on genus level were reported for horses under anesthetic for six hours, including an enrichment of the genera *Anaerostipes*, *Ethanoligenens* and *Enterococcus* (*Firmicutes*) 24 h later, while an enrichment of *Ruminococcus* (*Firmicutes*) was recorded after 48 h. However, further research is needed to gain more insights into anesthesia and its putative power to induce shifts within the equine intestinal microbiome.

Rapid proliferation of lactic acid producing bacteria is a feared consequence of a high starch diets, promoting lactic acidosis which is often followed by laminitis [136]. Interestingly laminitis was assumed to be associated with proliferation of streptococci [76], since earlier studies reported co-incidence [137, 138].

## Use of probiotics and their effects in horses

Recently, products classified as “probiotics” have reached the commercial market, not only for humans but also for horses. In 2001, experts of the World Health Organization (WHO) and Food and Agriculture Organization of the United Nations and the WHO (FAO/WHO) provided a very useful and actual definition of a probiotic: “live strains of strictly selected microorganisms which, when administered in adequate amounts, confer a health benefit on the host [139, 140]”. In the US, probiotics can either be classified as a drug needed to gain approval from the Food and Drug Authority (FDA) or as a feed supplement “generally regarded as safe (GRAS)” based on information provided by the producers, so they do not need to go through FDA approval [141]. In the European Union (EU), probiotics are regarded as feed additives and gut flora stabilizers for healthy animals [33]. The EU applies very strict regulations for products labeled as probiotics. Producers need to prove product identity, safety and efficacy to a scientific committee. Assessment and approval from the scientific committee and authorization under EU council regulation (EC) no. 1831/2003 on additives for use in animal nutrition is needed before market introduction [142]. In 2008, the EC no. 429/2008 provided detailed rules for the implementation of regulation 1831/2003. So far, bacteria such as *Lactobacillus*, *Enterococcus*, *Bacillus*, *Streptococcus* and *Bifidobacterium* are considered as putative beneficial probiotics for horses [141]. Probiotics should be able to survive the extreme gastric environment, have an antimicrobial property against pathogens and adhere to mucus and epithelial cells [143]. Probiotics for horses are designed to reach and establish themselves in the large colon, were many diseases occur. A recent study investigated the effects of multi-strain probiotics on the bacterial microbiota of foals during and after administration [144]. Limited changes were only found concerning relative abundance of bacterial families, with an enrichment of *Lactobacillus* in the probiotic group at week six [144]. Yet, evidence of probiotic efficiency in horses is weak despite several putative clinical applications including acute enterocolitis [145], diarrhea in foals [146] as well as fecal sand clearance [147].

## Future perspectives

Although microbiome research is considered an emerging science, with some areas of research still in their infancy, the field is progressing rapidly [148]. Nowadays, the most important research task is to gain a deeper understanding of the complex relationships between the gut microbiota, well-being and disease [149]. A meta-analysis of gut microbiome studies in humans revealed that some diseases are marked by the presence of potentially pathogenic microbes, whereas others are characterized by a depletion of health-associated bacteria [150]. Only recently, the first study investigating changes in the fecal microbiota using 16S rRNA gene data from microbiome analysis over a prolonged period (52 weeks) of healthy horses was published [106]. Throughout all seasons, *Firmicutes* and *Bacteroidetes* dominated the fecal microbiota, but supplementary forage, season and ambient weather conditions were significantly associated with change in the fecal microbiota composition [106]. These data provide an excellent starting point for further microbiome research investigating changes associated with metabolic disorders, infectious diseases or effects of drugs, since the first framework for a microbial composition associated with healthy horses has been set. However, disturbance of gut microbiota leading to or indicating illness still needs to be defined more precisely for horses.

Similar to the current trends in human medicine it might be possible to develop individual treatment opportunities for certain kinds of equine diseases which were marked through a certain and distinct pattern of microbial composition like equine grass sickness, laminitis or colitis. Moreover, fecal transplants are used to treat intestinal disorders including inflammatory bowel disease and recurrent *Clostridioides difficile* infections, and may eventually be used to treat a long list of disorders [151]. Besides technical questions associated with data generation and analysis, further research is needed to address the benefits and limits of different sampling sites for microbiome research in horses. Representativeness of different GIT sampling sites and feces have been discussed before, for example in pigs [152, 153]. A recent study on free-ranging bats revealed that the diversity and composition of intestine and guano samples differed substantially, likely reflecting the distinct processes that are known to occur in these microhabitats [154], as described above for different parts of the GIT in horses. Moreover, fecal samples retained more signal of host diet than intestinal samples, suggesting that fecal and intestinal sampling methods are not interchangeable [154].

As a further future perspective, research focused on effects of different antibiotics and/or application routes on the equine microbiome might reveal whether the absence or presence of certain key microbes is associated with drug-induced colitis. Currently, multi-drug resistance (MDR) in zoonotic bacteria such as *Escherichia coli* and *Staphylococcus aureus* are still a rising issue in equine medicine [155, 156]. Thus, further research might also identify dosages and application intervals for antibiotics which were not beneficial and sufficient for the horse patient alone, but also associated with a low selective pressure on resistant bacterial variants and thus hinder further accumulation of zoonotic MDR in horse clinics. In addition, metagenomics is currently considered as the most straightforward and affordable data that can be used to track transmission of strains [151], providing new perspectives to follow transmission routes of zoonotic bacteria.

## Conclusion

Our review summarizes the current understanding and progress in equine microbiome research (Additional file 1), which clearly is not yet at eyelevel with the latest vast progress in human medicine. Nonetheless, important first research initiatives have been kicked off, and fields worth investigating have been addressed clearly. Our review provides insights in commonly used techniques to explore the equine microbiome, their benefit and limitation as well as tools for data analysis. A smart combination of different techniques including the wet lab (Fig. 1) appears to be a good strategy to broaden and sustain the research outcomes.

## Supplementary information

**Additional file 1:**Overview on horse microbiome surveys

## Data Availability

Not applicable.

## Abbreviations

Bp: Base pair
EC: Council regulation
EU: European Union
FAO: Food and Agriculture Organization
FDA: Food and Drug Authority
GIT: Gastrointestinal tract
MALDI-TOF-MS: Matrix-Assisted Laser Desorption Ionization–Time of Flight Mass Spectrometry
MDR: Multi-drug resistance
NGS: Next-generation sequencing (NGS)
OUT: OPERATIONAL Taxonomic Units
QIIME: Quantitative Insights into Microbial Ecology
rRNA: Ribosomal ribonucleic acid
USEAR: Ultra-fast sequence analysis
WGS: Whole genome sequencing
WHO: World Health Organization
